# Community concepts of malaria-related illness with and without convulsions in southern Ghana

**DOI:** 10.1186/1475-2875-4-47

**Published:** 2005-09-27

**Authors:** Collins K Ahorlu, Kwadwo A Koram, Cynthia Ahorlu, Don de Savigny, Mitchell G Weiss

**Affiliations:** 1 Noguchi Memorial Institute for Medical Research, University of Ghana, Box LG581, Legon, Ghana; 2 Swiss Tropical Institute, Socinstrasse 57, CH-4002, Basel, Switzerland; 3 Department of Social Work, University of Ghana, Legon, Ghana

## Abstract

**Background:**

Malaria, both with or without convulsions, is a serious hardship for people living in endemic areas, especially in sub-Saharan Africa. Community references to malaria, however, may encompass other conditions, which was collectively designated malaria-related illness (MRI). Inasmuch as the presence or absence of convulsions reportedly affects timely help-seeking for malaria, a local comparison of these conditions is needed to inform malaria control.

**Methods:**

Vignette-based EMIC interviews (insider-perspective interviews) for MRI with convulsions (convulsion positive, MRI-CP) and without convulsions (convulsion negative, MRI-CN) were developed to study relevant features of MRI-related experience, meaning and behaviour in two rural communities in Ghana. These semi-structured interviews elicited both qualitative narrative and categorical codes for quantitative analysis. Interviews with 201 respondents were conducted.

**Results:**

The conditions depicted in the vignettes were well recognized by respondents and named with various local terms. Both presentations were considered serious, but MRI-CP was more frequently regarded potentially fatal than MRI-CN. More than 90.0% of respondents in both groups acknowledged the need to seek outside help. However, significantly more respondents advised appropriate help-seeking within 24 (p = 0.01) and 48 (p = 0.01) hours for MRI-CP. Over 50.0% of respondents responding to questions about MRI-CP identified MRI-CN as a cause of convulsions.

**Conclusion:**

Local comparison of MRI-CP and MRI-CN based on vignettes found a similar profile of reported categories of perceived causes, patterns of distress, help-seeking and preventive measures for both presentations. This differs from previous findings in sub-Saharan Africa, which assert communities regard the two conditions to be unrelated. The perceived relationships should be acknowledged in formulating strategies to control malaria through timely help-seeking and treatment to reduce childhood mortality.

## Background

According to the WHO about 1.3 million people died of malaria in 2003, and about 90% of these deaths occurred in sub-Saharan Africa [1-3]. Convulsions are features of many of these fatal illnesses, and caretakers easily recognise convulsions as a health problem in children. Though biomedical reasons for convulsions with malaria remain unclear, studies have shown important practical differences in local actions for managing childhood malaria-related illnesses (MRI) with convulsions (i.e. convulsion positive, MRI-CP) and without convulsions (i.e. convulsion negative, MRI-CN) [4,5]. Many studies argue that convulsions, often considered unrelated to MRI, lead to a significant alteration in the meanings of the illness experience and behaviour, indicating more frequent reliance on traditional healers as the primary source of treatment or in combination with biomedicine [4,6-12].

Practical questions remain about how this cultural epidemiology of MRI-CP relates to timely, appropriate health-seeking by caretakers. Many studies have reported that MRI-CN is generally treated first at home – either with modern pharmaceuticals (mainly analgesics and inadequate antimalarials), or herbal medications, or both – and caretakers seek outside help only when the illness persists or when they observe a high fever [8,10,13-16]. A recent report by de Savigny *et al*. [14], on the other hand, found that for about 80% of malaria-related deaths in Tanzania, modern help was used first. Most control programmes focus on various clinical features of malaria apart from convulsions, and few studies have compared community views of MRI-CP and MRI-CN. Strategies for malaria control assume that appropriate interventions at the febrile stage of the disease will prevent progression to more serious, life- threatening illness.

To explain locally perceived relationships between MRI-CP and MRI-CN, and to consider their practical implications for local management and timely, appropriate help-seeking, this study compares local illness experiences, meaning and behaviour associated with the two conditions. It aims to clarify their common and distinctive feature, and to consider implications for local management and timely, appropriate help-seeking. Based on the framework for local classification, the study examines the assertion that they are locally perceived to be two distinct conditions, as reported in the literature [6-9].

### Study Area

This study was conducted from October, 2002 to April, 2004 in two malaria-endemic villages, Galo-Sota in the Keta District and Obosomase in the Akuapim North District of Ghana. Keta District is located in the coastal savannah vegetation zone of the Volta region, where about a third of the total surface area is covered with lakes and ponds. The district has a population of 137,751 (Government of Ghana national population census, 2000), consisting mainly of the Anlo patrilineal descent group (98.8%). The Anlo language is one of the closely related dialects spoken among the Ewe-speaking people of Ghana (Keta District Annual Report, 2001). The Anlo people are predominantly subsistence food-crop farmers, but they also cultivate shallot (a tropical spice grown in commercial quantities). Some are fishermen and petty traders. Galo-Sota is a rural village with a population of about 6,000 to 7,000. A health post is situated at the centre of the village, staffed by a nurse/midwife, two community nurses and two auxiliary workers. Malaria was the most common health problem treated at the community health post in 2002. A tributary of the Volta River passes through the village, demarcating a boundary between Galo and Sota, which together constitute Galo-Sota.

The Akuapim North District in the eastern region of Ghana is situated in the forest zone. The district population is 113,915, according to the last census (National population census, 2000). The Akuapim-Twi-speaking people, members of the Akan matrilineal descent group, predominate in the district. These Akuapim people are also mainly food-crop farmers and petty traders. Oil palm, a cash crop, is cultivated on a limited scale. The district is currently being prepared as a designated site for a malaria vaccine trial. Among health science activities in the area is a Centre for Scientific Research into Plant Medicine (Akuapim North District Annual Report, 2001). Obosomase (population 7,000 to 8,000) is the rural study village in the Akuapim North District. It has a community clinic staffed by a nurse/midwife, a community health nurse and one auxiliary staff. As in Galo-Sota, malaria was the most common health problem treated at the community clinic in 2002.

### Study Methods

Two EMIC interview tools were developed locally to study the relationship between sociocultural factors and appropriate treatment-seeking for children up to five years of age. EMIC interviews are locally adapted instruments for assessing representations of illness or specified health problems from the perspective of affected persons, their family or community members. They elaborate the distribution of local insiders' illness-related experience, meaning and behaviour integrating qualitative and quantitative approaches [17,18]. The designs of these semi-structured vignette-based interviews were informed by baseline ethnographic data which generated illness narratives that specified locally relevant MRI-related categories of distress, perceived causes and help-seeking behaviours. The vignettes for MRI-CP and MRI-CN are included in an appendix "a" and "b". To account for gender differences, two vignettes were presented with men responding to male vignettes and women responding to female vignettes.

Respondents aged 20 years or more were selected randomly from community registers to participate in the interviews. However, younger respondents between the ages of 20 and 25 years of age were unwilling to participate in the interview using the vignette depicting MRI with convulsion, which introduced some age difference between the two groups of respondents. The reason for refusal was, they claimed, that they did not have any experience to talk about. Informal discussions, however, revealed that some people believed that if they talked about convulsions, this might bring that condition to their children. This attitude was observed in both communities. One hundred interviews were conducted using vignettes depicting MRI-CN and 101 using vignettes depicting MRI-CP. Respondents were evenly distributed by sex and community. Few of those originally selected eventually refused to participate in either of the interviews – two men and one woman at Obosomase and three men at Galo-Sota. The dissenting individuals were replaced from the registers.

Interviews were conducted by the first author in the local languages, and data were recorded by a research assistant with a degree in sociology, who was trained to record both qualitative and quantitative interview data. The EMIC instrument was pre-tested to refine it and gain experience. The pretesting showed it was unnecessary to tape record the interviews, inasmuch as the data were recorded very well by the research assistant.

Data from the two communities were pooled for this analysis. Item-specific qualitative narrative data were entered into a word processor (Microsoft Word) and imported in a structured format for automatic coding, referencing text segments of EMIC interview items in MAXqda, a programme for textual analysis [19]. These data were analysed to complement and elaborate the quantitative accounts and to clarify relevant aspects of illness-related experience, meaning and behaviour. Variables of interest in the quantitative data-base were imported into MAXqda as selection variables. This integrated approach to quantitative and qualitative analysis enabled us to perform a phenomenological analysis of relevant coded segments from selected respondents to categories that were analysed with quantitative methods.

Quantitative data were double entered in DOS EpiInfo version 6.04 and subsequently analysed with the EpiInfo Windows version 3.3 [20]. The frequencies of spontaneously reported and probed responses were examined to specified categories of cultural epidemiological variables for patterns of distress (PD), perceived causes (PC), self-help at home (SH), outside help-seeking (HS) and preventive measure (PC). The prominence of reported variables (2 = spontaneously reported, 1 = probed and 0 = not reported) was compared in the MRI-CP and MRI-CN groups using the Wilcoxon test. The percentage reporting each category and the fraction reporting the category spontaneously are presented in tables. Respondents were also asked to identify the most troubling symptom (PD) and the most important perceived cause (PC). These variables were compared in the two groups with Fisher's exact test. The variables, as initially coded, were also grouped and analysed under relevant subgroups, based on investigators' judgement of shared meanings under a broader heading.

Reported duration from illness onset to observe, wait or treat at home before seeking-help from outside the home for the children presented in the two vignettes was analysed to compute anticipated timely, appropriate help-seeking for MRI-CP and MRI-CN within 24 and 48 hours. The reported timeliness was then compared for the two vignette groups.

## Results

The socio-demographic characteristics of respondents are presented in Table 1. Households were headed mainly by men, 75.2% and 67.0% in MRI-CP and MRI-CN groups respectively. Many respondents could not specify their household income, which was about the same in the two groups of respondents.

**Table 1:** Demographic characteristics of respondents

**Demographic characteristics of respondents**	**Vignettes presented**	
	
	**With convulsion (N = 101)**	**Without convulsion (N = 100)**
Age of respondents*		
Mean age	49.0	38.0
Std. Dev.	15.8	12.5
Mode	35	25
Female: male ratio	1:1	1:1
**Education**		
Mean years of education	6.1	6.6
No education (%)	22.9	19.0
Highest education (years)	20	14.0
**Marital status (%)**		
Married	66.3	58.0
Never married	3.0	20.0
Divorced/separated	16.8	16.0
Widowed	13.9	6.0
**Religion (%)**		
Christianity	55.4	64.0
Traditional religion	40.6	35.0
Islamic	3.0	1.0
Others	1.0	0.0
**Household income (%)**		
Regular & dependable	23.8	23
Uncertain/not sure	52.5	48
Not regular & dependable	23.7	29

* P < .01 (T-test)

The children presented in the two vignettes were the focus of interview questions. Local names or terms used to describe their illnesses and their approximate English translations or meanings are presented in Table 2. The two conditions presented were identified by different names and terms. All the terms had been identified during ethnographic study as local terms for MRI-CP and MRI-CN.

**Table 2:** Local terms and their approximate English equivalents

**Vignette with convulsions**			
**Obosomase**	**English translation**	**Galo-Sota**	**English translation**

Sroakyereno	Attacked by the sky	Xeivitsoe	Taken/attacked by a bird
Adiatorniso	Obsessed or possessed	Adukpodzidor dzedzi	Attacked by garbage dump illness
Atridii barima akyereno	Attacked by male malaria	Hehedor dzedzi	Attacked by stretching illness
		Dordzagla/dorsese dzedzi	Attacked by a strong illness
		Dzifotorwotsoe	Taken by the people of the sky
		Miatorwotsoe	Taken by our friends

**Vignette without convulsions**^1^			

Atridii	Hot body, yellowish urine, yellowish eyes, Vomiting, cold, and shivering, bodily pains, weakness, refusal of food, easily startled, paleness, weight loss, etc	Asra	Hot body, yellowish urine, yellowish eyes, Vomiting, cold and shivering, bodily pains, weakness, refusal of food, easily startled, paleness, weight loss, etc
Ebun		Fever	
Feve		Nudza	
Malaria		Malaria	

^1^Local terms and names for MRI without convulsions have no single equivalent in English, and were used interchangeably to represent similar conditions. "Malaria" and "fever" have also been incorporated in local usage as terms and names.

Respondents in the MRI-CP group appeared somewhat more likely than MRI-CN respondents to consider the condition "usually fatal" (39.6% against 27.0%). Narrative elaborated concerns about mortality: "*This kind of illness kills children easily, especially if parents do not respond quickly to treating the child*."

*"As for this problem, it is a very serious one. If it does not kill the child then it could destroy the child's ability to reason properly*."

For MRI-CN children, mortality was likely to be associated with some vulnerability or neglected treatment: *"This is a very serious problem; so long as the child is still very small she may die if not treated properly and early*."

"*Fever can kill children because people do not consider the disease serious when it is starting, so before they realize it, it has already become worse for the child. What I mean is that it is not a condition that should usually kill, but if it is not seen and treated early, it does kill*."

In both situations, a majority of respondents (98.0% for MRI-CP and 96.0% for MRI-CN), said it was necessary that someone stays at home to care for the sick child. The mother of the child was the obvious choice for 97.0% of MRI-CP and for 95.0% of the respondents for vignette depicting MRI-CN.

### Patterns of distress for childhood MRI with and without convulsions

Reported symptoms of both conditions are presented in Table 3. Some of these were distinctive for each presentation, and others were reported for both presentations, but with some differences. C*onvulsion-related *symptoms were more prominent in the MRI-CP group, and more frequently reported as most troubling. On the other hand, MRI-CN respondents reported significantly more *fever-related *symptom and identified them more frequently as most troubling. *Non specific *symptoms, except for breathlessness, were also most prominent in responses of the MRI-CN group.

**Table 3:** Reported symptoms and single most troubling symptom of MRI with and without convulsions

**Categories of distress reported**^1^	**Reported spontaneously and probed**^2^				**Most troubling**	
	
	**With Convulsion (n = 101)**		**Without convulsion (n = 100)**		**With Convulsion (n = 101)**	**Without convulsion (n = 100)**
			
	**Total (%)**	**Fraction Spont.**	**Total (%)**	**Fraction Spont.**		
**Convulsions related symptoms**	**98.3**	**0.72**	**64.0**	0.05**	**67.3**	6.0**
Unconscious	86.2	0.39	18.0	0.06**	26.7	2.0**
Stiffness	x	x	0.0	0.00	21.8	0.0**
Easily startled/frightened	76.3	0.32	59.0	0.03**	7.9	3.0
Rolling the eye balls	x	x	0.0	0.00	5.9	0.0**
Biting the lips	42.5	0.17	0.0	0.00**	2.0	0.0
Foaming mouth	55.4	0.31	0.0	0.00**	2.0	0.0
Folded arms	52.5	0.64	0.0	0.00**	1.0	0.0
Shaking	x	x	0.0	0.00	0.0	1.0

**Fever related symptoms**	**37.6**	**0.55**	**98.0**	0.79**	**9.9**	34.0**
Hot bodies	x	x	x	x	6.9	11.0
Sweating	14.0	0.14	55.0	0.06	1.0	0.0
Yellowish eyes	28.8	0.65	97.0	0.78**	1.0	18.0**
Yellowish urine	x	x	x	X	1.0	4.0
Chills and Rigors	x	x	x	X	0.0	1.0

**Non specific symptoms**	**93.1**	**0.52**	**99.0**	0.66**	**20.8**	56.0**
Weakness	72.3	0.52	86.0	0.51*	11.9	15.0
Breathlessness	51.4	0.14	29.0	0.07**	5.9	1.0
Refusal of food	x	x	x	X	1.0	16.0**
Weight loss	35.5	0.14	63.0	0.25**	1.0	12.0
Diarrhoea	0.0	0.00	33.0	0.06**	1.0	0.0
Vomiting	x	x	x	X	0.0	7.0**
Paleness	44.5	0.20	74.0	0.23**	0.0	4.0
Sleepiness	0.0	0.00	12.0	0.25**	0.0	1.0
Bitterness in the mouth	0.0	0.00	33.0	0.61**	0.0	0.0
Joint and bodily pains	16.0	0.25	36.0	0.22**	0.0	0.0
Crying	x	x	x	x	0.0	0.0

^1 ^Symptoms analysed as groups (in bold) based on reported categories that follow.

*p ≤ 0.10, ** p ≤ 0.05. Wilcoxon test for comparison of prominence of reported categories (2 = spontaneous, 1 = probed response, 0 = not reported), and Fisher's exact test for most troubling symptoms.

^2^Column values indicate frequency of reported categories and the fraction of these reported spontaneously. Column values marked by "x" indicate categories specified in the vignettes.

The following narratives indicate how people explain the symptoms presented in the vignettes. A woman speaking about convulsive illness characterized typical features: "*In most cases, the child's jaws are locked, and the child becomes very stiff. Also, foaming fluid comes from the mouth. In some cases the child becomes unconscious*."

Another respondent explained, "*Among most typical symptoms, the child begins to shiver and all of a sudden becomes very stiff and hot, and rolls the eye balls*."

A man explained typical symptoms for MRI-CN: "*The first sign is that the child feels very cold and then at certain times feels hot. It is also possible that the child loses weight and sweats so much. The child's eyes also turn yellow because of the fever. The colour of his urine will look yellowish, and he may become very weak*."

A woman explained, "*The child's body becomes hot and this makes her cry a lot. She may complain of headache, and feels cold and shivers. She may also find it difficult to eat; her eyes become yellow, and she looks pale and weak*."

Most troubling symptoms reported more frequently by MRI-CP respondents included *unconsciousness *and *stiffness*. For MRI-CN respondents, most troubling symptoms more frequently reported were *yellowish eyes*, *refusal of food*, and *weight loss*. Weakness was identified by some respondents in both groups as most troubling.

Among features of distress, apart from somatic symptoms of malaria, a number of problems were reported to affect the families of the children in the vignettes with and without convulsions (as specified by percent reported/ fraction spontaneous). Nearly all respondents reported loss of income for families of the affected children (99.0% reporting/0. 42 fraction spontaneous for MRI-CP, and 100% reporting/0.46 fraction spontaneous for MRI-CN), *concern about the course of illness *(100.0%/0.90 MRI-CP, and 99.0%/0.90 MRI-CN) and *sadness, anxiety or worry *(99.0%/0.94 MRI-CP, and 100%/0.93 MRI-CN). However, *financial concerns *(unavailability of funds for treatment and inability to work for money), which leads to anxiety, were frequently reported as the most troubling categories of distress for the family, (71.3% for MRI-CP, and 76.0% for MRI-CN). *Concern about the course of illness *was also considered one of the most troubling categories of distress for the family (26.7% for MRI-CP, and 21.0%% for MRI-CN). These were common features of both groups without statistically significant difference between them.

The following representative narratives explain the importance of income loss to the family of a child with MRI. An MRI-CP respondent elaborated: "*Generally, if a child becomes ill, the parents are worried because they do not know what could happen to their child, and in addition to that, it could lead to financial problems for the family*." Another respondent lamented: "*The child's condition can adversely affect her in the future, so the parents would become so bothered and worried about the child, especially if they do not have money. The child could become deaf and mute or have a mental problem. I have seen one like that before*."

Referring to MRI-CN, a man said: "*Fever kills so the family would be worried. Also money issues could be a problem for them*."

Another respondent explained: "*It is very normal that when your child is sick, you become worried, especially if you do not have money*."

### Perceived causes

The prominence of many perceived causes differed for the two groups (Table 4). The most frequently reported perceived causes of convulsions reported by MRI-CP respondents were *spirits*, *phlegm*, worm infections and *atridii/asra/malaria; *this last category refers to local terms for MRI as a cause of convulsions. Most frequently reported perceived causes of MRI by MRI-CN respondents were *mosquito bites*, *eating too much fatty or oily food *and *heat from the sun*.

**Table 4:** Reported perceived causes and the single most important cause for MRI with and without convulsions

**Perceived causes reported**^1^	**How reported**^2^				**Most important**	
	
	**With convulsion (n = 101)**		**Without convulsion (n = 100)**		**With convulsion (n = 101)**	**Without convulsion (n = 100)**
			
	**Total (%)**	**Fraction Spont.**	**Total (%)**	**Fraction Spont.**		
**Insect bites**						
Mosquito bite	44.4	0.24	94.0	0.48**	6.9	35.0**

Infections & malaria-related	**95.1**	**0.64**	**73.0**	0.07**	**43.6**	1.0**
Phlegm	77.1	0.41	24.0	0.04**	15.8	0.0**
*Atridii/asra/malaria*	57.4	0.54	0.0	0.00**	13.9	0.0**
Worm infections	58.2	0.17	68.0	0.06	13.9	1.0**

**Exposure**	**39.6**	**0.67**	**89.0**	0.63**	**9.9**	33.0**
Heat from the sun or fire	27.8	0.75	81.0	0.68**	8.9	31.0**
Airborne/exposure	16.9	0.41	22.0	0.14	1.0	2.0

**Sanitation & hygiene**	**58.4**	**0.63**	**90.0**	0.51**	**10.0**	**11.0**
Personal hygiene	36.8	0.78	51.0	0.65*	5.0	4.0
Sanitation	25.9	0.73	30.0	0.73	4.0	4.0
Playing on the ground	21.9	0.46	43.0	0.37**	1.0	0.0
Houseflies	34.7	0.14	78.0	0.13**	0.0	3.0*

**Supernatural**	**84.2**	**0.35**	**79.0**	0.04**	**19.8**	1.0**
Spirits	78.3	0.38	63.0	0.02**	19.8	1.0**
Evil eyes or sorcery	50.4	0.24	41.0	0.00**	0.0	0.0

**Food and drink**	**36.7**	**0.38**	**96.0**	0.41**	**2.0**	18.0**
Eating unbalanced diet	0.0	0.00	17.0	0.12**	2.0	3.0
Fatty/oily food	26.8	0.15	84.0	0.12**	0.0	8.0**
Eating unripe fruits	12.9	0.00	72.0	0.08	0.0	6.0**
Impure water	11.8	0.83	31.0	0.39**	0.0	1.0

^1 ^Perceived causes analysed as groups (in bold) based on reported categories that follow.

*p ≤ 0.10 and ** p ≤ 0.05. Wilcoxon test for comparison of prominence of reported categories (2 = spontaneous, 1 = probed response, 0 = not reported), and Fisher's exact test for most important perceived causes.

^2^Column values indicate frequency of reported categories and the fraction of these reported spontaneously.

Overlapping meanings were also reported in respondents' accounts of perceived causes. For example, a woman in the MRI-CP group explained: "*Some people claim that if children play on refuse dumps they easily get convulsion, but I also think that a child can get it through mosquito bites, because these give fever, which can lead to this condition. Worms can also cause a child to get this condition, because worms release some substances into the child's stomach, which in turn gives the child phlegm. And this can cause a convulsion. Evil spirits can also cause children to have this illness*."

A man said: "*Malaria is the major cause, but in some cases, spiritual forces can also cause a convulsion. It also depends on the kinds of food that children take, like unbalanced diet*."

Explaining MRI-CN, a woman commented: "*This condition could have been caused by worm infestations or houseflies that perch on food and contaminate it before it is eaten. Sometimes too mosquito bites can cause it*."

Another said: "*Maybe she wasn't eating good food. Bad food like fatty/oily foods can lead to this problem. I know that mosquito bites or living in a dirty environment could also cause it. And exposure to the heat from the sun can also cause it*."

### Self-help at home

Respondents reported various self-help options for both conditions. The most prominent among reported sources of self-help for MRI of both types was herbal-based remedies for drinking (Table 5). Among pharmaceutical medicines, purchasing drugs from the drug or chemical shops was most prominent in responses of both groups, but significantly more so for the MRI-CN group. Other traditional remedies, such as scarification were reported more for the MRI-CP vignette.

**Table 5:** Self-help at home for MRI with and without convulsions

**Self help at home reported**^1^	**How reported**^2^			
	
	**Vignette with convulsion (n = 101)**		**Vignette without convulsion (n = 100)**	
	
	**Total**	**Fraction Spont.**	**Total**	**Fraction Spont.**
**Herbal & other local actions**	**96.0**	**0.92**	**99.0**	0.76**
Home-prepared herbal medications for drinking	72.3	0.75	83.0	0.60
Bathing with ordinary cold water (tepid sponging)	67.3	0.34	70.0	0.34
Home-prepared herbal medications for enema	52.4	0.62	44.0	0.46*
Home-prepared herbal medications for bathing	40.6	0.73	33.0	0.30*
Other actions (scarification etc)	22.9	0.96	5.0	1.00**
Home-prepared herbal medications for other uses	8.0	0.88	8.0	0.50

**Modern pharmaceuticals**	**52.5**	**0.34**	**92.0**	0.60**
Drug/chemical shops to purchase drugs	47.6	0.31	89.0	0.57**
Other leftover drugs at home	7.7	0.26	9.0	0.33
Leftover antimalarials at home	1.0	1.00	7.0	0.86**

^1 ^Self-help at home analysed as groups (in bold) based on reported categories that follow.

*p ≤ 0.10 and ** p ≤ 0.05. Wilcoxon test for comparison of prominence of reported categories (2 = spontaneous, 1 = probed response, 0 = not reported).

^2^Column values indicate frequency of reported categories and the fraction of these reported spontaneously.

A woman emphasized the value of various traditional remedies for MRI-CP: "*In some cases they say that water kept in a 'banku pot' (utensil for preparing a local maize meal) overnight, mixed with urine, can be used to bathe the child for relief. Herbal preparations can also be used to bathe the child. Some people prefer to prepare some herbs for the child to drink. Some also give honey, while some people may buy drugs from the chemical sellers*."

Commenting on MRI-CP vignette, a man indicated how various interventions might all be appropriate: "*Some of the possible actions may be to buy drugs from a chemical seller for the child to drink or prepare herbs for drinking or enema*."

A respondent in the MRI-CN vignette group indicated the value of diverse treatment for that condition as well: "*Some medicine could be given at home as a measure to reduce the severity of the illness. The medicine could be bought from the drug stores if there are no leftovers. Some herbal medications can also be given to the child*."

Another said: "*The family may decide to buy drugs for the child; they could also decide to prepare herbs for the child to drink*."

### Outside help-seeking

The need to seek help outside the home was reported by nearly everyone for both conditions (Table 6). More MRI-CP respondents, however, were concerned about getting treatment right away. In the MRI-CP group, 29.0% said outside help should be sought within 24 hours, compared with 9.0% for the MRI-CN group (p < 0.01). The same relative priority was indicated by responses advising treatment within 48 hours (53.0% MRI-CP and 34.0% MRI-CN, p = 0.01).

**Table 6:** Help-seeking from outside the home for MRI with and without convulsions

**Outside help seeking reported**^1^	**How reported**^2^			
	
	**Vignette with convulsion (n = 101)**		**Vignette without convulsion (n = 100)**	
	
	**Total**	**Fraction Spont.**	**Total**	**Fraction Spont.**
**Clinic & hospital**	**99.0**	**0.96**	**100.0**	**1.00**
Government/community clinic	95.1	0.96	99.0	0.97
Government hospital	95.0	0.96	98.0	1.00

**Local healers & religious leaders**	**84.2**	**0.40**	**66.0**	0.26**
Local healers	69.4	0.46	57.0	0.26**
Religious leaders (pastors/Imams)	44.3	0.11	35.0	0.06

**Other source of modern pharmaceuticals**	**13.8**	**0.43**	**19.0**	**0.21**
Health worker in the community for advice	12.8	0.38	17.0	0.12
Drug/chemical shops for advice	3.0	0.33	8.0	0.25

^1 ^Outside help-seeking analysed as groups (in bold) based on reported categories that follow.

*p ≤ 0.10 and ** p ≤ 0.05. Wilcoxon test for comparison of prominence of reported categories (2 = spontaneous, 1 = probed response, 0 = not reported).

^2^Column values indicate frequency of reported categories and the fraction of these reported spontaneously.

Similar outside sources of help were identified by respondents in both groups (Table 6). Although more MRI-CP respondents recommended traditional healers, other health care providers were suggested by similar percentages from both groups.

Commenting on outside help-seeking for MRI-CP, a woman explained that home remedies at some point were not enough: "*If the herbs used at home do not work other people who know more herbs, like traditional healers, could be consulted. The child could also be taken to the clinic or hospital, but this costs money*."

Another observed: "*As soon as it is clear that what is done at home does not work, the child would have to be sent to a clinic or hospital. The hospital or clinic is the best place, and the family must go, but they can also see a traditional healer for treatment*."

Some MRI-CN respondents like this woman, compared modern and traditional health care providers favourably: "*A clinic or a hospital should be the best places to go but some traditional healers also know about herbs that work*."

A man emphasized the importance of not waiting too long before getting help from a doctor: "*As soon as the child's condition does not get better after the home treatment, the family should consult a doctor*."

### Prevention

Most respondents in both groups (74.3% MRI-CP and 84.0% MRI-CN) said the conditions in the vignettes could have been prevented, there was, however, a borderline significant difference (p = 0.06). Categories of preventive measures suggested for both presentations were similar (Table 7). Preventive measures frequently reported included *preventing mosquito bites*, *staying less in the sun*, *maintaining personal hygiene*, *environmental cleanliness*, *drinking clean water*, *avoiding fatty or oily foods*, and *reducing strenuous or hard work/play*. These were reported more frequently by respondents in MRI-CN group. Preventive measures based on magico-religious ideas were mentioned more frequently in the MRI-CP group. Measures frequently reported by similar percentages in both groups included *taking medications regularly *(herbal or biomedicine) and *deworming children regularly*.

**Table 7:** Preventive measures for MRI with and without convulsions

**Preventive measures reported**^1^	**How reported**^2^			
	
	**Vignette with convulsion (n = 101)**		**Vignette without convulsion (n = 100)**	
	
	**Total**	**Fraction Spont.**	**Total**	**Fraction Spont.**
**Insect bites**				
Prevent mosquitoes' bite^3^	58.4	0.27	94.0	0.31**

**Regular medications**	**88.1**	**0.24**	**87.0**	**0.27**
Taking herbal or biomedicine regularly	75.4	0.45	73.0	0.44
Deworming regularly	50.4	0.18	49.0	0.16

**Sun & strenuous play**	**33.6**	**0.21**	**80.0**	0.29**
Stay less in the sun or near fire	31.9	0.22	79.0	0.28**
Reduction in strenuous play	8.0	0.13	35.0	0.20**

**Sanitation & hygiene**	**55.4**	**0.66**	**71.0**	0.66**
Cleaning the environment	29.7	0.63	49.0	0.74**
Keeping personal hygiene	51.5	0.65	66.0	0.67**

**Food and drink**	**59.4**	**0.47**	**86.0**	0.38**
Eating balanced diet	33.6	0.59	40.0	0.63
Avoid fatty/oily foods	29.8	0.03	63.0	0.14**
Drinking clean water	18.8	0.37	32.0	0.41**
Eating less starchy food	17.8	0.11	28.0	0.11*
Eating on time (not going hungry for long)	10.9	0.00	16.0	0.13
Drink a lot of vegetable soup	10.9	0.28	18.0	0.28

**Magico-religious**	**47.5**	**0.19**	**37.0**	0.00**
Avoid offending evil spirits like the witches	23.8	0.25	15.0	0.00*
Attend to ancestral spirits and family gods	40.7	0.20	32.0	0.00

Don't know/cannot tell	22.8	1.00	14.0	1.00

^1 ^Preventive measures analysed as groups (in bold) based on reported categories that follow.

*p ≤ 0.10 and ** p ≤ 0.05. Wilcoxon test for comparison of prominence of reported categories (2 = spontaneous, 1 = probed response, 0 = not reported).

^2^Column values indicate frequency of reported categories and the fraction of these reported spontaneously.

## Discussion

This study identifies similarities and differences between local concepts, meanings, self help at home, help-seeking from outside the home and recommended preventive measures for MRI with and without convulsions. As reported in many studies across sub-Saharan Africa, spiritual forces dominate perceived causes for MRI-CP, compared to MRI-CN [4,6,8,9,14,22-24] However, Findings reported here show that priority of timely, appropriate care is not reduced by local traditional perceived causes of convulsive illness. Although more respondents in the convulsions group reported magico-religious causes than respondents in non-convulsions groups, they also more frequently recommended medical treatment within 24 and 48 hours of illness onset.

Although concerns about supernatural causes of convulsions were evident, even affecting our ability to recruit young adult respondents for the MRI-CP sample, most respondents for the two presentations reported that the children in the vignettes should be taken to the clinic or hospital for treatment. This differs from many reports across sub-Saharan Africa, where studies emphases use of traditional healers as the primary source of treatment for convulsions, rather than modern medical care [6-9,21]. Some studies however, have reported the use of both biomedicine and traditional healers [10,11,22]. Findings are consistent with those of de Savigny *et al*. [14] in Tanzania, reporting that 78.7% of fatal malaria cases received modern treatment as the first resort for their last illness episode.

The 28.0% of MRI-CP respondents recommending appropriate treatment within 24 hours was significantly more than the 9.0% of MRI-CN respondents who did so (p < 0.01), but the rates for both are relatively low, and far below the designated Abuja target. The percentages were higher for 48 hours (53.0% and 34.0%, p = 0.01), but still lower than the 60.0% specified in the Abuja target for 24 hours (15). This could mean that the message of the priority of timely treatment has either not yet reached these communities or it is not compelling enough to motivate action and hence needs reinforcement.

It is notable that despite the distinctiveness in many studies of MRI-CP and MRI-CN illnesses, 57.4% of respondents in the MRI-CP group have reported that malaria is a cause of convulsions, and 13.9% said malaria is the most important cause of convulsions. The emerging local awareness of the link between mosquitoes, malaria and convulsions should be strengthened to reinforce the priority of timely, appropriate treatment for febrile malaria without convulsions. Information, education and communication (IEC) have important roles to play in that regard. More people acknowledge the value of home-based treatment for MRI-CN, consistent with a policy to promote that option for uncomplicated malaria. Reliance on traditional healers, especially for MRI-CP, however, remains a problem, inasmuch as this results in many children not receiving timely antimalarial treatment. The use of a mix of traditional remedies (herbal and rituals) and biomedical treatments are consistent with the literature [5,10-12,22].

For both conditions, findings also suggest approaches to prevention that are related to local perceived causes, mostly involving avoidance of identified causes. Most people, similar in both groups, recommended regular medications as a preventive measure, indicating favourable prospects for implementing intermittent preventive treatment (IPT) for pregnant women. The finding suggests it may be feasible to introduce intermittent preventive treatment for children under five years of age to reduce morbidity and mortality in this vulnerable age group. When evaluating such policy options, the risk of drugs being used inappropriately should be weighed against prospects for reducing mortality.

Widespread recommendations to avoid mosquito bites to prevent both conditions indicate good prospects in these communities and others like them for acceptance of insecticide-treated bednets. The idea that uncomplicated malaria may progress to convulsions may further reinforce such an approach to prevention. These issues would need emphasis when developing IEC as an intervention to reduce MRI-related morbidity and mortality in the study communities.

The study shows that despite the complexity of local experience, meaning and behaviour with respect to malaria-related illnesses, it is possible to identify the distribution of categories and explain local illness behaviours, their sociocultural determinants, and practical implications in endemic local rural communities. Relating results to timely help-seeking and malaria prevention suggests ways of incorporating local relevance ideas into the design and implementation of local programme strategies, especially IEC, an indication of how cultural epidemiology may inform malaria control activities to make them sustainable to reduce MRI-related morbidity and mortality.

Though, this study was carried out in two rural communities in southern Ghana findings may be generally applicable in most part of Ghana, especially the southern half of the country. However, local variations must be considered when interpreting findings for areas outside the study localities.

## Competing interests

The author(s) declare that they have no competing interests.

## Authors' contributions

CK Ahorlu was involved in the conception and design of the study, fieldwork, data management, analysis, interpretation and writing of this paper.

KA Koram was involved in the conception and design of the study, and data management.

C Ahorlu was involved in the conception of the study, fieldwork, and data management.

D de Savigny was involved in analysis and interpretation of data, and writing of this paper.

MG Weiss was involved in the conception and design of the study, data analysis, interpretation and writing of this paper.

## Ethical Review

The study was approved by the institutional review boards of Noguchi Memorial Institute for Medical Research and the Swiss Tropical Institute. It was also reviewed by the WHO/TDR Ethical Review Committee.

## 

**Table 8 - Appendix 1a.** 

**Appendix 1a. vignette depicting MRI with convulsion positive (MRI-CP)**	
**Introduction to vignette **'I appreciate your agreeing to talk to me about a problem that affects many children in this district. I want to understand how you think about it. Keep in mind that it is your ideas that I am interested in, so please do not feel there is a right or wrong answer to the questions I will ask you. Do not be shy to tell me what you think. So then, let me tell you a story about a child called Kofi/Ama who has this problem.'	
MALE 'Kofi is a 2/12 year-old boy who had been feeling fine and playing happily. One day last week, Kofi woke up crying, and his mother found that his body felt very hot. Kofi seemed to be feeling cold and he was shivering. This was on and off for some time and he refused to eat anything. His urine was yellow in colour. He has vomited too. A few hours later, he started rolling his eyes. With his eyes opened wide, he was shaking and became stiff'	FEMALE 'Ama is a 2/12 year-old girl who had been feeling fine and playing happily. One day last week, Ama woke up crying, and her mother found that her body felt very hot. Ama seemed to be feeling cold and she was shivering. This was on and off for sometime and she refused to eat anything. Her urine was yellow in colour. She vomited too. A few hours later, she started rolling her eyes. With her eyes opened wide, she was shaking and became stiff'
Appendix 1b. Vignette depicting MRI with convulsion negative (MRI-CN)	
**Introduction to vignette **'I appreciate your agreeing to talk to me about a problem that affects many people in this district. I want to understand how you think about it. Keep in mind that it is your ideas that I am interested in, so please do not feel there is a right or wrong answer to the questions I will ask you. Do not be shy to tell me what you think. So then, let me tell you a story about a child called Kofi/Ama who has this problem.'	
'Kofi is a 2/12 year-old boy who had been feeling fine and playing happily. One day last week, Kofi woke up crying, and his mother found that his body felt very hot. Kofi seemed to be feeling cold and he was shivering. This was on and off for sometime. He refused to eat anything. His urine was a yellow colour, and after a few hours, he vomited.'	'Ama is a 2/12 year-old girl who had been feeling fine and playing happily. One day last week, Ama woke up crying, and her mother found that her body felt very hot. Ama seemed to be feeling cold and she was shivering. This was on and off for sometime. She refused to eat anything. Her urine was a yellow colour, and after a few hours, she vomited.'
